# The Venerial Diseases, Including Stricture of the Male Urethra

**Published:** 1880-05

**Authors:** 


I.—THE VENERIAL DISEASES, INCLUDING STRICTURE OF THE MALE URETHRA. By E. L. Keyes, A.M., M.D., Prof. of Dermatology and Adjunct Prof. of Surgery in the Bellevue Hospital Medical College; one of the Surgeons of Bellevue Hospital; Consulting Surgeon to Charity Hospital, etc , etc. New York. WM. WOOD & Co.; 27 Great Jones street. 1880.

